# Exploring the Analgesic Potential of L-Lysine: Molecular Mechanisms, Preclinical Evidence, and Implications for Pharmaceutical Pain Therapy

**DOI:** 10.3390/pharmaceutics17050666

**Published:** 2025-05-19

**Authors:** Walaa Alibrahem, Nihad Kharrat Helu, Gréta Törős, Csaba Oláh, József Prokisch

**Affiliations:** 1 Doctoral School of Health Sciences, University of Debrecen, Egyetem Tér 1, 4028 Debrecen, Hungary; nihad.kharrat.helu@mailbox.unideb.hu (N.K.H.); olahcs@gmail.com (C.O.); 2 Institute of Animal Science, Biotechnology and Nature Conservation, Faculty of Agricultural and Food Sciences and Environmental Management, University of Debrecen, Böszörményi Street 138, 4032 Debrecen, Hungary; toros.greta@agr.unideb.hu (G.T.); jprokisch@agr.unideb.hu (J.P.); 3 Doctoral School of Animal Husbandry, University of Debrecen, Böszörményi Street 138, 4032 Debrecen, Hungary; 4 Mathias Institute, University of Tokaj, Eötvös Str. 7, 3950 Sárospatak, Hungary; 5 Neurosurgery Department, Borsod County University Teaching Hospital, Szentpéteri Kapu 72-76, 3526 Miskolc, Hungary

**Keywords:** L-lysine, chronic pain, analgesia, NMDA receptor inhibition, serotonin modulation, dopamine, fibromyalgia, pain management, neurotransmission

## Abstract

Pain is a complex, multifaceted sensory–emotional state. It still poses a significant challenge in clinical treatment, especially in cases of chronic pain. Concerns associated with the use of opioids as analgesics have led to the search for new and safer pain relievers. This review examines the potential of lysine in pain control by exploring its molecular mechanisms and the preclinical evidence and clinical implications. Lysine has demonstrated analgesic effects by inhibiting NMDA receptors, modulating dopamine and serotonin pathways, and interfering with neuroimmune signaling cascades. Studies in animal models have shown that the administration of lysine reduces pain responses without altering motor function. Despite the favorable profile of lysine in terms of minor side effects and its promising effectiveness as a nutritional supplement, more research is needed to optimize its efficacy, adjust its dosage, and ensure its safety for long-term use.

## 1. Introduction

Pain serves as more than simply an unpleasant experience—it has an evolutionary purpose in living organisms and has been recognized as the “fifth vital sign” [1]. Pain is a multifaceted experience with both physical and emotional components [1]. The physical aspect of pain reflects the presence of a harmful stimulus or the possibility of an injury occurring; encompasses pain’s sensory and discriminatory qualities; and identifies pain’s location [2]. The emotional qualities of pain, however, include suffering, fear, and anxiety [3]. Pain’s emotional qualities also serve a telling function [4]. They motivate and are involved in particular thoughts and behaviors designed to avoid the stressful situation or threatening stimulus perceived [3,4]. Pain has a biological purpose in that it maintains homeostasis, detects potential danger, and enhances survival [1]. In reality, there can be considerable variability in the experience of pain between individuals and even within the same person [5]. It is this adaptive role of pain that becomes dysfunctional in chronic pain states in which pain signals persist longer than is protective. Pain is broadly categorized into two types: acute and chronic [2]. This division is convenient but artificial, as shown in Table 1. There are necessary differences between these two types of pain, nonetheless. The complex issues of pain examined here, such as neuropathic pain, are only beginning to be understood among medical professionals, never mind the general lay public [5]. Within the dominant biomedical construct of pain, there are three accepted main categories, each with its own subcategories: nociceptive, neuropathic, and psychogenic [4]. Medical professionals therefore usually regard this type of pain using a straightforward cause-and-effect paradigm [5]. This is a problem rooted in the inability to pinpoint any specific cause of pain, as well as difficulties in promoting patient adherence to multifaceted and multimodal pain control strategies [2]. It is especially troubling as a growing body of evidence points to significant interactions between central nervous system processes and how people experience pain; effectively, specialist interventions and treatments are rendered less effective [6]. Pain control frequently involves both pharmacological and non-pharmacological approaches [5,6]. The referred methods may include analgesics, invasive treatments, physical therapy, behavioral therapy, and exercises [4]. Hormonal and dietary adjustments are also an option that has been practiced in many locations, but increasing opioid epidemics and problems have rekindled the quest for alternatives [6]. The need for alternative, safer, and more effective pain treatments has grown in importance in recent years [5]. The rise in abuse, addiction, and overdose associated with opioids has drawn significant attention to the challenges of comfortable and safe pain treatments in healthcare [5].

**Table 1:** Comprehensive comparison between acute and chronic pain.

Category	Acute Pain	Chronic Pain	References
Definition	A sudden, unpleasant sensory and emotional experience due to injury or surgery; short duration.	Persistent or recurrent pain lasting longer than 3–6 months, often without ongoing tissue damage.	[1,2]
Duration	Short-term (days to weeks).	Long-term (months to years).	[3]
Biological Function	Adaptive and protective; serves as a warning signal to prevent further harm.	Often maladaptive; becomes a pathological state with no protective benefit.	[3]
Causes	Acute injury, surgery, burns, or acute inflammation.	Caused by chronic diseases (e.g., arthritis, neuropathy) or sometimes idiopathic (unknown cause).	[4]
Emotional Components	Usually mild, linked to transient anxiety.	Prominent; includes depression, chronic anxiety, and psychological suffering.	[5]
Response to Treatment	Generally responsive to conventional pharmacologic treatments like analgesics and NSAIDs.	Often limited responses; requires multidisciplinary approaches (medical, psychological, physical).	[4,5]
Impact on Quality of Life	Minimal and temporary disruption in daily functioning.	A major impact on the physical, emotional, and social aspects of life.	[5]
Functional Consequences	Rarely causes long-term disability; usually resolves with healing.	Leads to ongoing functional limitations and disability (e.g., mobility, work, social activity).	[6]

### The Current Pain Management Strategies

Pain is a protective mechanism that occurs as part of the body’s response to injury, procedures, disease, chemotherapy, and infection. However, pain may become abnormal when it outlives its purpose and prevents healing or is excessively severe [6]. Pain control and management are an important part of any medical treatment [7]. Pain management strategies can be classified into pharmacological methods, non-pharmacological methods, or a mixture of both [5]. Despite a notable improvement in pain control medications, their side effects limit their use in some cases [4]. A multidrug approach is frequently needed to attain effective pain control. Attention has been directed to alternative and additional therapies and resources, called ‘adjuvant therapeutics’ [2]. For example, pain control strategies can focus on therapies other than medicine [7]. These can include physical treatment; occupational treatment; mental healthcare treatment; biofeedback; relaxation techniques; changes in one’s way of living; complementary medicine, including acupuncture, chiropractic care, homeopathy, and massage; and dietary improvements [7]. Though some studies have reported good results, others have been less successful. The effectiveness of these strategies has been debated and viewed to differ based on the model of pain. Currently, even when the traditional pain control therapies are successful, pain persists in a significant number of patients [8]. This has led not only to continuing interest in scientific investigations into basic mechanisms, more practical pain treatment methods, and new therapeutic targets but also to a growing engagement in pain management improvements in special ethnic or geographic areas. Although one of the main amino acids, lysine, is reported to be involved in analgesia, its potential has not been assessed in comprehensive publications on pain management, suggesting additional studies to find new methods or to improve the existing methods for pain control [8].

## 2. A Basic Overview of Lysine

**Figure 1:**
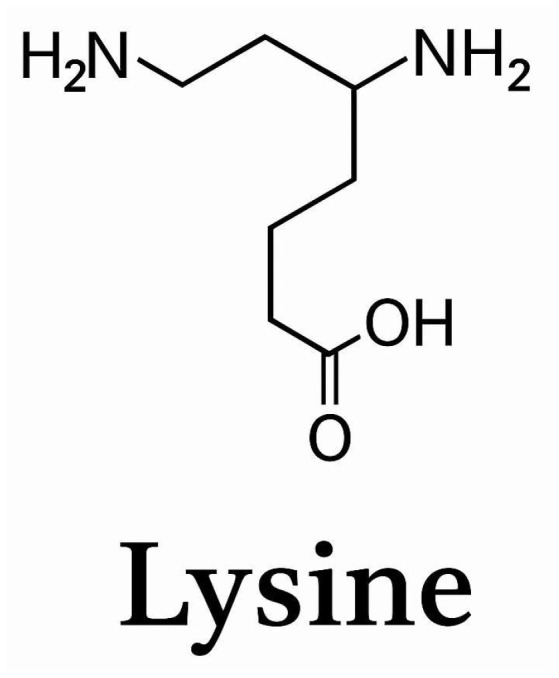
Chemical formula for lysine.

Lysine is a basic essential amino acid, appearing as white crystals or powder, as shown in Figure 1. It is stored in the muscle, skin, bones, and tendons, mitigating their breakdown and rebuilding; similarly, it is stored in organs like the liver [9]. It assists in calcium absorption and maintains its reserves, which are essential components of maintaining blood and bone health [10]. Overall, it is essential in building body mass and plays a significant role in growth mechanisms due to it being the strongest amino acid responsible for protein synthesis [11]. Building proteins cannot be performed without their presence; proteins are the basis of the body and present in all cells of the body; they are necessary for growth, development, and health maintenance and are responsible for the repair and regeneration of damaged/destroyed tissues [12]. Since these body mass proteins consist of the enzymes and hormones necessary for usable nutrients and their transfer via the bloodstream to the muscles, the presence of this amino acid enables proteinase formation, also consisting of enzymes; Proteinases are enzymes that digest proteins in food, breaking them down into amino acids that the body can use [10].

The second role of this amino acid in the body is supporting collagen production. Since tissue proteins are made up of amino acids, this suggests that body tissues are primarily composed of proteins—making the presence of this amino acid essential for building and repairing these tissues; wounds and the skin’s connective tissue cannot heal in afflicted states without their constant consumption [11]. Still, this fact demonstrates its possible useful properties in chronic and postoperative pain. While this amino acid itself cannot be consumed for collagen, the amino acid is necessary in its production [13]. Lysine can also help to alleviate tissue-damage-related pain for these aforementioned reasons. In addition to all of this, conventional medicine and dietetics, on the subject of chronic pain in comparison with muscle pain, suggest the presence of this amino acid as a necessary part of one’s diet for joint pain caused by damaged tissues [14]. Furthermore, on pain and lysine, the recommended daily intake level for lysine in adults is 30–40 mg/kg; lysine has calming properties, as it is a serotonin antagonist; studies suggest its consumption for anxiety and stress. Still, since 60 mg/kg of this amino acid causes toxicity, it is advised to consult a therapist when using this substance [13,14].

### Physiological Roles and Lysine Deficiency

It cannot be synthesized from simpler compounds in animals, including humans, and it has to be consumed in the diet [9]. Lysine is an essential amino acid, used not only for the production of proteins but also hormone-like signal substances, antibodies, and other molecules involved in the immune response [15]. Lysine deficiency affects the host by reducing weight gain and feed efficiency, as well as impairing many metabolic functions, while limiting immune responses. Lysine deficiency depresses food intake and growth with extreme lean tissue loss, whereas weight loss with an energy deficit causes a metabolic adaptation involving nitrogen savings [16].

Deficiencies of essential amino acids like leucine, isoleucine, valine, threonine, phenylalanine, tryptophan, methionine, and lysine have toxic effects [17]. In ruminants, interference with their carbohydrate metabolism occurs, while in humans and poultry, it leads to intellectual disability, growth retardation, and high levels of ammonia in the blood [15]. Lysine deficiency can be particularly serious in pigs. It severely limits weight gainand feed efficiency and impairs the body’s ability to resist pathogens [17].

## 3. Lysine and Its Implications for Pain

Lysine is an essential amino acid, one of the most hydrophilic, docking at α-helical membrane proteins or polar phospholipids; it is also a precursor molecule in synthesizing certain fatty acids. Various neurotransmitter receptors interact with extracellular lysine [9]. Temporary, selectively altered permeability in specific regions of the nerves, affecting certain fatty acids but not lysine, results in a brief increase in the levels of free lysine within the peripheral nervous system (PNS), suggesting that lysine may play a role in the early phases of transient pain states [18]. Moreover, pain associated with strong physical damage, particularly neuropathic pain, is accompanied by a slight increase in the lysine content in the peripheral blood [19]. Nutritional lysine reduces the generation of chronic pain, prolonging the latency until the appearance of a persistent painful state in some injuries. After chronic constriction injuries in the tooth pulp, the time elapsed until the beginning of unevoked nociceptive hypersensitivity was approximately doubled when using lysine [18]. A weakly positive, but not significant, effect was observed in rats with peripheral entrapment injuries. Under strict dietary conditions, the group supplemented with lysine showed a slightly adverse effect on the improvement of pain recovery following peripheral compression [19]. Restricted diets represent the main natural conditions able to alter lysine metabolism. Among the products of muscle proteolysis, muscle damage can lead to the release of lysine, resulting in a local increase in its levels in the peripheral blood. Under dietary lysine restrictions, the increased availability of free lysine in the blood could be part of the early local response to nerve injury, which would favor the generation of acute pain and hamper the development of chronic neuropathy [20]. On the other hand, acute pain of various origins usually causes a transient rise in systemic blood free lysine. This amino acid also acts at the brain level in some psychiatric disorders, in particular in anxiety, by acting as a partial serotonergic receptor antagonist [21]. One possible explanation for this is that Lysine-sensitive gastrointestinal absorption mechanisms, which are pH- or ion-dependent, can be transiently altered by a lysine-deficient diet [16]. Lowered lysine levels in the peripheral blood, which normally occur after physical nerve damage, could be part of the failure of the early systemic response to the injury that enhances and prolongs the generation of spontaneous ectopic activity in the primary afferents. The physiological consequence of dysfunction in the serotonergic system is an increase in pain sensitivity. Some psychoactive drugs may promote pain relief by acting as serotonergic enhancers [20].

### 3.1. The Mechanisms of Action of Lysine in Pain Control

**Figure 2:**
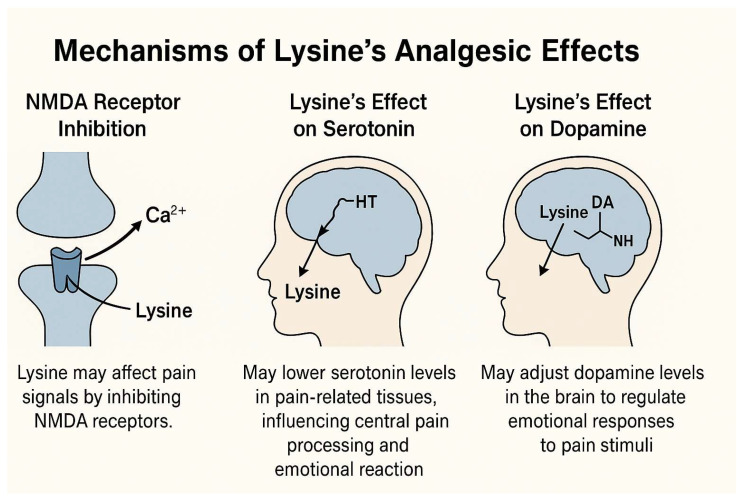
Potential mechanisms of action of lysine in pain perception and modulation [21,22,23].

Pain or analgesic sensations are defined as unpleasant sensations that occur as a signal of actual or potential harm to the body [3]. However, the perception and modulation of pain are very complicated biological functions that involve various peripheral tissues, signaling cascades, and biochemical processes in the nervous system [4]. To date, several potential mechanisms of pain perception and modulation have been proposed [21]. Among the potential mechanisms of pain signal perception, the N-methyl-D-aspartate (NMDA) receptor is localized in the pain pathway and is critical to the induction and maintenance of long-term potentiation in the pain pathway [22]. Because lysine inhibits NMDA receptors, it can potentially interact with pain signal perception via NMDA receptors [23]. Among its possible effects, lysine may decrease the serotonin concentrations around pain-related tissues, as shown in Figure 2. Serotonin is known to regulate the central processing of harmful stimuli in the brain, as well as modulating emotional responses to harmful stimuli [24]. Alternatively, it can be hypothesized that programmatic lysine influences emotional responses to harmful stimuli by modulating dopamine levels at the brain’s pain signal reception sites, helping to regulate excessive emotional reactions [8].

### 3.2. The Inhibition of NMDA Receptors

**Figure 3:**
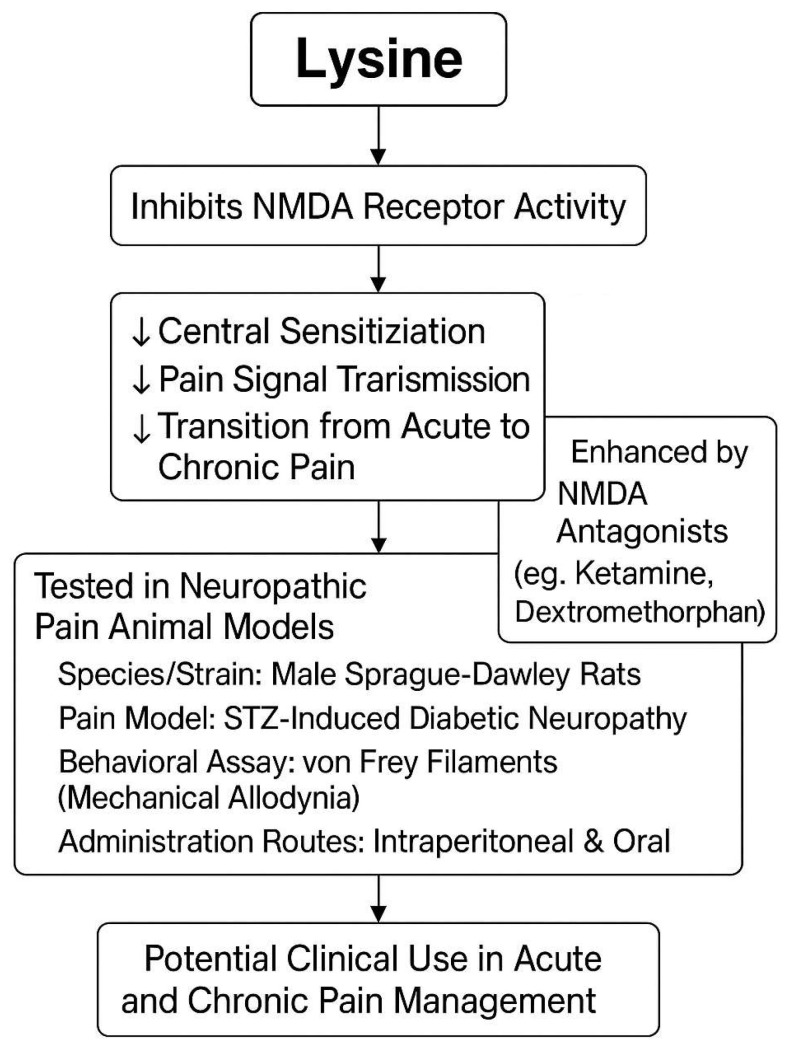
Lysine’s role in pain relief via NMDA receptor inhibition [39,40].

NMDA receptors (NMDARs) have been widely studied for their critical role in processing pain, especially in the cortex and the limbic structures of the brain. Activation of the NMDARs is important for the induction of central sensitization and the creation of long-lasting chronic pain conditions [25]. The inhibition of NMDARs in the central nervous system represents an important target for drugs intended to alleviate neuropathic pain, as shown in Figure 3. Evidence has shown that lysine can act as a competitive inhibitor of NMDAR activity and can inhibit NMDAR-evoked currents in the dorsal root ganglion (DRG) neurons [26]. In other studies, the intrathecal administration of lysine was seen to induce analgesia in rats through the inhibition of the NMDARs by preventing the phosphorylation of components involved in NMDA receptor signaling [27]. Thus, lysine may also act as an NMDA antagonist in the central nervous system, and the accumulation of this amino acid, previously reported in the plasma, could exert antinociceptive effects [28]. If lysine indeed has further inhibitory effects on NMDAR activity, as proposed above, it may be utilized for pain control [29]. This could provide a mechanistic explanation for the promising results with lysine in clinical studies of both acute and chronic pain alleviation [30]. This potential inhibitory effect of lysine on the NMDA receptors is proposed to be the underlying biological mechanism for the previously reported analgesic effects of lysine in both chronic and acute pain states [29]. This putative function of lysine could bring novel insights into chronic pain treatment and could be explored further in future studies to optimize the formulation and dosage of lysine to maximize its therapeutic efficacy [31].

NMDA receptors (NMDARs) have received considerable attention in basic science research on pain control, in part because they contribute to central sensitization, which is thought to relate to the conversion of acute into chronic pain [32]. Several commonly used pharmaceutical interventions for pain target these receptors, including the NMDAR antagonists ketamine and dextromethorphan [33]. Interestingly, lysine is reported to inhibit nociception, and this ability is acquired through spinal NMDARs [34]. According to one study employed an animal model of neuropathic pain to explore the possible role of NMDARs in the antinociceptive action of lysine, a common constituent of foods and drugs, following its systemic administration via two different routes [35]. Focusing on their paw-licking behaviors in response to thermal stimulation, it was found that lysine produced a dose-dependent analgesic effect in rats with painful peripheral nerve injuries. Analgesia is consistent with a decreased frequency of action potential in the post-synaptic dorsal horn neurons. Importantly, these two findings of decreased action potential and flick responses are potentiated up to 100% if rats are pretreated with the systemic NMDAR antagonists ketamine or dextromethorphan [36]. No treatment has physiological effects on injured animals per se. This line of evidence supports the hypothesis that lysine exerts its antinociceptive action through the inhibition of spinal NMDARs following its systemic administration. An understanding of the inhibitory action of lysine in the NMDARs provides a rationale for its previously demonstrated ability to reduce pain sensations [37]. In the field of clinical pain management, lysine may demonstrate broader potential for managing both acute and chronic pain conditions [38]. However, further investigations are needed to validate these inferences and to optimize the lysine regimens for improved clinical pain control [38].

### 3.3. Modulation of Serotonin and Dopamine Levels

Pain perception is influenced by the modulation of serotonin and dopamine levels. Ninety-five percent of serotonin is found in the bowels. Its production and release from the enterochromaffin and enterochromaffin-like cells are amino-acid-dependent [41]. Serotonin also comes from the diet: cheese, cold cuts, dairy, turkey, chocolate, pasta, cereals, etc. Some viewpoints propose decreases in serotonin as having a role in triggering migraines. Another study discussed how subjects with “high interpersonal sensitivity” and low D-serine plasma concentrations are more sensitive to sharp pain [42].

**Figure 4:**
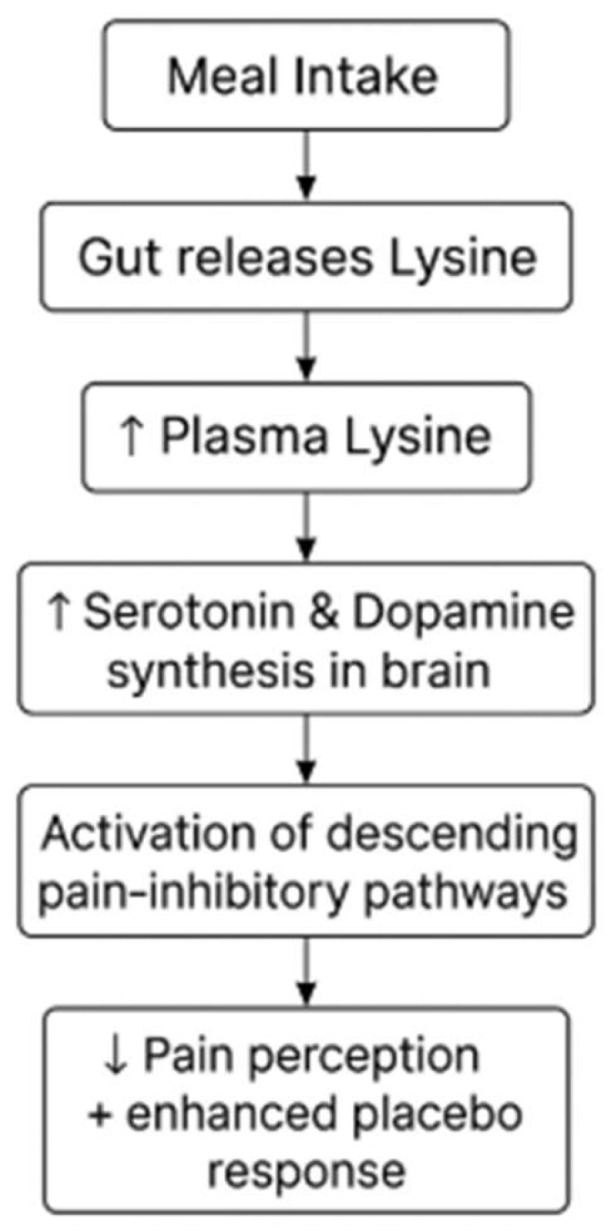
Lysine-mediated biochemical pathways for pain reduction.

Low levels of serotonin may alter the affective and sensory components of pain processing, rendering nociceptive inputs more salient and facilitating their access to attention and short-term perceptual and memory systems [43]. A decrease in top-down inhibition may also add to an augmented sensitivity to pain-related information [44]. Serotonin and dopamine activate the pain-inhibitory descending pathways to the dorsal horn of the spinal cord, leading to the inhibition of pain [42]. It was recently found that the gut stores lysine and releases it into the portal circulation in response to a meal, raising plasma lysine concentrations and enhancing serotonin production [43]. Changes in the perception of pain can be modulated by amino acids, such as lysine, which increase the synthesis of serotonin and dopamine, as shown in Figure 4. The administration of lysine could increase the serotonin and dopamine production in the brain, leading to a reduction in the experiences of pain from the sensory cortex and enhancing the placebo amplification of events in the emotional cortex. The retention of conscious perceptions of pain under a single sub-acute stressor could be lessened by simultaneously enhancing central serotonin levels [45].

Thus, the concomitant presence of stress and biochemically increased serotonin may better prevent the buffering effects of distraction on pain awareness. Decreased plasma tryptophan and increased plasma tyrosine have significant therapeutic implications for enhancements in pain control and improving mood disorders through the administration of synthetic amino acids [44]. Decades after the first controlled study on the analgesic potential of placebo administration, it was shown that the effect of the placebo was mediated by this endogenous brain opioid [46]. Experimental manipulation of the endogenous opioid alters the placebo’s efficacy for clear experimental pain, linking emotional and top-down cognitive mechanisms to the efficacy of the placebo [47]. Using amino acid supplements, such as lysine, to enhance the serotonin and dopamine production in the brain may be a novel way to control the pain emanating during purification and other clinical settings, as well as improving the stand-alone enhancements of pharmacological and psychological interventions [48]. Advancements in our biochemical understanding could lead to the manipulation of such effects and contribute to the effective management of pain, as shown in Table 2.

**Table 2:** Key factors influencing pain modulation through neurotransmitter pathways.

Factor	Role in Pain Modulation	References
Serotonin	↓ levels linked to increased pain sensitivity, migraines, and reduced top-down inhibition	[42]
Dopamine	Activates pain-inhibitory descending pathways	[43]
Lysine	Enhances serotonin and dopamine production; reduces pain perception	[48]
The Gut–Brain Axis	Lysine released post-meal enhances serotonin synthesis in the brain	[45]
Amino Acids (Tryptophan/Tyrosine)	Influence neurotransmitter balance, with implications for mood and pain control	[44]
Placebo and Opioid System	Pain relief via endogenous opioids, linked with top-down emotional and cognitive factors	[47]

Lysine can reach the central nervous system (CNS) and cross the blood–brain barrier (BBB) via large neutral amino acid transporters, resulting in less accumulation within the mesolimbic nuclei than other substrates [49].

In patients with schizophrenia treated with lysine in addition to risperidone or haloperidol, clinical trials have shown no worsening in their psychotic symptoms. This is likely due to the continuous occupancy of the D2 receptors by these antipsychotics. Haloperidol, as a typical antipsychotic, binds D2 receptors with high affinity and in a sustained manner, while risperidone, though atypical, still provides sufficient receptor occupancy to buffer against transient increases in dopamine levels [50].

## 4. Clinical Studies on Lysine for Pain Management

Since 1979, laboratory research has indicated that the basic amino acid lysine possesses pain-relieving efficacy when administered parenterally [46]. An intravenous dose of lysine as hydrochloride salt produces a powerful, long-lasting, and dose-related reduction in pain sensitivity in ‘neuropathic’ and ‘non-neuropathic’ animals [51]. As compared to non-experimental bases, its analgesic effect is prompt and long-lasting. In non-human species, both acute responses to noxious stimulation and pain of anemic, inflammatory, thermal, chemogenic, and neuropathological origins are affected. Histological and behavioral data indicate that lysine interacts with the activity at the opioid receptor complex, facilitating rather than blocking it. Further studies are needed to determine the frequency and level of such interaction [2]. In 1981, animal research suggested that lysine had potential efficacy in humans. Employing a controlled, double-blind design, 26 healthy human volunteers were assembled, and their sensitivity to thermal pain was tested before and after the administration of lysine or a placebo [52]. Lysine was found to be highly significant in increasing both the temperature necessary to produce pain and the time it took for the onset of this pain [53]. Furthermore, maximal differences between lysine and the placebo were observed over the full 180 min post-injection test period, suggesting a large effect size [52]. Follow-up studies are required to determine the optimal dose and method of administration with which the impact of lysine on sensitivity to other forms of pain is hastened [15]. There have been rigorous, completed clinical studies involving lysine as a pain management tool and the experience of lysine as a pain medicine since 1981 [53].

### 4.1. Animal Studies

Animal experiments in pain models provide consistent evidence that treatment with lysine has an antinociceptive effect [54]. For example, in the rat formalin test, the subcutaneous administration of 0.5 or 2 g/kg of lysine significantly inhibited the second phase of paw-licking time without affecting the locomotor activity of the animals [55]. The rats in the lysine group displayed fewer formalin-induced biphasic licking behaviors [56]. In addition, lysine significantly reduced the number of c-Fos immunoreactive neurons in the dorsal horn of their L4–L6 spinal cord sections at either 0.5 or 2 g/kg [57]. In an acetic acid writhing model in mice, 1 g/kg of lysine completely suppressed the writhing produced by 0.6% acetic acid, and an antagonistic effect on the anti-writhing effect of morphine was revealed when the mice were pretreated with 3 g/kg of L-arginine for a continuous period of 6 days [15]. In other work, rats were administered 5% acetic acid intraperitoneally to induce writhing [58]. Lysine (0.1, 0.3, 0.5, 0.6, 1, 1.5, 2, 3, or 5 g/kg, s.c.) was injected partly in one of two sets of experiments: 4 min prior to acetic acid (a 24 h test group) or using a 3-day treatment, with the first administration given 4 h after acetic acid (a 72 h test group) [59]. The number of writhes was significantly reduced by the treatment with lysine at a dose of 5 g/kg for the 24 h test group [60]. However, The antinociceptive efficacy of lysine was reversed by pretreatment with a nitric oxide synthase substrate, the L-arginine derivative ADMA, but not by the L-arginine precursor [60]. This is the first study to demonstrate antinociception in formalin and writhing tests using lysine treatment and that this effect may be mediated by nitricergic pathways. Table 3 summarizes the most important points related to experimental studies in rats. Major strengths of these data include the consistent antinociceptive effects of lysine in multiple pain tests performed in several independently run experiments and in two species [58]. The existing studies to date have shown the efficacy of multiple doses of lysine in acute or chronic pain treatment models [59]. Given that lysine is a known inhibitory transmitter in the spinal dorsal horn that has previously been shown to modulate pain, these data suggest that lysine in the brain may be a potential target for the management of clinical pain [23]. Efforts are needed to elucidate the reported analgesic mechanisms of lysine further and to determine the most effective and safe drug delivery mode according to patient feedback. Given that animal data are not conclusively predictive of the pain relief in humans due to species differences in receptor genetics and distribution, care should be taken to develop and translate future studies into clinical trials [57].

### 4.2. Human Clinical Trials

A total of 24 participants (23 women and 1 man), with a mean age of 47.1 years (range: 19–74), completed one trial. Most of these patients had not used any fibromyalgia medication prior to the survey. Three patients had used low doses of tricyclic drugs, and two patients had used an SSRI. Most of the patients had been diagnosed with fibromyalgia for several years, with a mean diagnosis duration of 96 months [61]. The mean duration of their pain experience was 145.1 months. The clients were randomized to receive L-lysine (2.64 g a day), ascorbic acid (57 mg a day), or a placebo in a double-blind, crossover design for three months. There was a one-month-long placebo-free period between the treatment periods. The sequence of administration of the placebo and the active treatment was random [62]. Clients participated in three study visits: at the baseline, after three months of treatment (the first and active treatment period) or after two months of treatment (the second and active treatment period), and after three months of the other treatment (the placebo period). None of the patients dropped out of the study after starting the study medication. It must also be noted that only some fibromyalgia drugs have similar adverse gastrointestinal effects to those of L-lysine. The medication treating against the symptoms of fibromyalgia consists mainly of simple analgesics, non-steroidal anti-inflammatory drugs, norepinephrine/serotonin reuptake inhibitors, and, to a lesser extent, tricyclic drugs and GABA analogs. The lysine treatment schedule was based on previous publications describing the treatment for herpes simplex infections: individual doses of 1–3 g and a total daily dose of 8–40 g of L-lysine [63]. This design may show the maximum pain-reducing effect, particularly if all three doses prevent the development of the pain stimulus. Due to the material used, the study design could not incorporate three doses, as the individual doses were relatively high (2.64 g), nor could this dose be divided into consumption five times a day. The results of this study indicated that L-lysine and ascorbic acid supplementation could have a role in the management of chronic pain. In this study, the majority of the clients experienced significantly reduced pain whilst they were using lysine, although others reported a slight increase in the occurrence of pain. The clients who responded well to the treatment reported a mean reduction of 21.8% in their pain experience. In addition, a general pain threshold was tested. The clients could be divided according to their reactions into four categories: those who experienced no effect (29%), those experiencing an increase (11%) or a decrease (52%) in the pain threshold, and those whose daily average experience of pain over 7 days changed from day to night time or vice versa (8%). Such a diverse response to the ingestion of lysine has previously been noted in individuals treated for herpes simplex infections [48].

## 5. L-Lysine: Pharmacological Profile and Clinical Applications in Pain Management

L-lysine exhibits minimal pharmacokinetic interactions with conventional analgesics, likely due to its high absorption efficiency via specific transporters and its lack of interference with plasma protein binding [64]. Oral doses of L-lysine, within a range of 300 mg to 6 g daily, show a clinically acceptable level of safety [65]. Clinical evidence indicates that lysine derivatives, particularly in the form of salts such as lysine-essinate, lysine-clonixinate, and ketoprofen-lysine, have demonstrated remarkable efficacy in relieving pain due to various conditions, including radiculopathy, osteoarthritis, and post-surgical pain [64]. These compounds are commonly These compounds are commonly used in combination with non-steroidal anti-inflammatory drugs (NSAIDs), such as acetylsalicylic acid, or in the form of lysine salts to improve its absorption, reduce gastric irritation, and achieve a faster onset of action [66].

One notable aspect of these combinations is their ability to reduce the doses of opioids or NSAIDs required when they are used together without causing adverse pharmacokinetic interactions [67]. This synergistic effect is attributed in part to L-lysine’s ability to modulate inflammatory responses via peripheral mechanisms, in addition to its potential central effects through the modulation of microglial activity and regulation of neurotransmitter pathways such as those for serotonin and dopamine [55]. However, despite the relatively good safety profile of these formulations, the use of L-lysine in sensitive clinical populations, such as psychiatric patients, requires some caution given the potential for a mild effect on the central neurotransmitters, which may interfere with neuropsychiatric stability in these patients [64].

## 6. Potential Side Effects and Safety Considerations with Lysine

A potentially important consideration when administering high doses of lysine is that they are commonly associated with mild diarrhea [16]. The introduction of ready-to-use fortified spread (RUSF) represents a sustainable long-term intervention that can support the most vulnerable and impoverished families, particularly in cases where, in addition to TB medication, treatment for other concomitant conditions—such as pain management—is also required [68]. An inadequate intake of lysine is unlikely in a normal diet that includes meat, fish, eggs, soy, nuts, and legumes. The upper safety level has been stated to be an oral intake of 40 g/day [16]. Allergic reactions are considered unlikely at the current intake levels of lysine [69]. Red yeast rice lysine is a new patented extract obtained from the cultivation of Monascus sp. strains of yeast on rice, containing high amounts as compared to other plant sources [68]. L-lysine has been supplemented into the diet of schizophrenic patients treated with antipsychotic drugs. The literature confirms the absence of information or knowledge on possible allergic effects or other modes of action with adverse effects [16]. Likely vulnerable groups have been identified with concern. In what are considered appropriate, prudent, and transparent risk assessments, There has been increased attention on the need for rigorous monitoring of the introduction of L-lysine–fortified products in Scandinavia [70]. Additionally, for acute inflammatory pain, it may be beneficial to engage multiple pathways simultaneously [59]. Since lysine may inhibit constitutive nitric oxide synthesis and seems to have far fewer side effects, it may also be particularly important as part of a combination treatment with COX inhibitors for acute inflammatory pain, as indicated in Table 4 [71]. The idea is to gain control over the rapid molecular switches that provoke neuropathic pain without using NSAIDs. Finally, as lysine might be antagonistic to drugs intended for mental illness, caution is called as regards the introduction of lysine into patients of this kind [70].

**Table 4:** Summary of potential side effects and safety considerations with L-Lysine.

Category	Findings and Considerations	Reference
Common Adverse Effects	Mild gastrointestinal disturbances (e.g., diarrhea) have been reported at high oral doses.	[16]
Average Daily Intake (Europe)	Average intake in Europe: 2.6 g/day (women), 2.9 g/day (men); significantly below the RDI (8.4–10.4 g/day).	[72]
Recommended Daily Intake (RDI)	Oral intake of 8.4 and 10.4 g/day is considered safe in healthy individuals.	[16]
Clinical Use	Up to 6 g/day orally is well tolerated in schizophrenic patients without adverse interactions with antipsychotic drugs.	[62]
Contraindicated	Caution is advised for individuals with mental health conditions; children with PKU have shown tolerance to lysine.	[71]
Pharmaceutical Interventions	Lysine may enhance pain control when combined with COX inhibitors and may reduce the reliance on NSAIDs.	[71]

## 7. Future Directions in the Research on Lysine for Pain Management

The future directions of research into lysine’s role as an adjunct in pain management have been reviewed. Several emerging trends in pain research that might promote the incorporation of lysine have been identified.

Possible future directions and novel approaches in the research into lysine as an adjunct in pain management have been explored. The first is that the use of lysine may spur the development of new technologies and methodologies that the pain field could adopt, such as in assessing the impact of personalized therapies through genotyping, as has been applied in pharmacogenomic pain research trials involving opioids [2]. Second, as lysine consumption increases and research into lysine grows, several compounds used as lysine supplements have not been studied in depth, and their stability and bioavailability have not been determined [16]. A third area of research will expand the currently biochemistry- and pharmacology-focused research to include studies on the clinical impact and applications of lysine. These will include studies on behavior, pharmacology, and clinical interventions to determine in more complex ways how lysine and its impacts will intersect. Interdisciplinary research, as a necessity and common methodology in the fields of biology and psychology, will be pursued [73].

The second area of exploration involves suggestions for possible future directions and prioritization of research into the role of lysine as an adjunct in pain management [55]. The first recommendation for future studies is that many of the existing trials that have been conducted have been short-term trials looking at the analgesic effects of lysine, but it is not yet known how lysine may influence broader outcomes of pain conditions such as stress, anxiety, depression, or sleep quality. Thus, the consideration that long-term studies on lysine as an adjunct for pain management may be beneficial and may yield a broader impact constitutes an important avenue of research that has not yet been widely explored. The second recommendation is that as the impact of lysine on pain continues to be elaborated, lysine may be able to serve as a means of dietary intervention in the field of public health to significantly reduce the economic and personal health burdens of chronic or acute pain. Cross-field research on lysine as a public health intervention is encouraged as a way to link public and political interests with scientific health research.

### Emerging Trends and Technologies

With the increasing size of pain populations, pain research has now shifted from the general level to the individual level by applying genomics, proteomics, metabolomics, and other advanced methodologies [74]. Nutrigenomics research may provide new insights into lysine. Also, with the development of big data and artificial intelligence (AI), there is a trend of intelligentizing lysine research, such as using digital monitoring methods and Internet medical models [73]. Hereby, it is proposed that the transformation and application of new technologies and new methods are critical to enhancing the value of lysine’s utilization. At present, digital health technologies, such as e-health and telemedicine, are becoming more and more important. It is worth considering the combination of lysine supplementation and digital health technologies to conduct more precise and dynamic research. Equally, traditional oral drug delivery systems still have various deficiencies, such as excessive irritation to the gastrointestinal tract, low bioavailability, and short durations. The development of new drug delivery systems could overcome these shortcomings and enhance lysine’s effectiveness. For example, micro-needle painless patch administration technology could allow lysine to be absorbed by the skin, and sustained-release technologies could maintain blood concentrations of lysine. Additionally, chronic pain (neuropathic pain) accounts for 20–25% of the pain population. It has been found that the pathological mechanism of chronic pain is different from that for acute pain, and the same treatment can have very different effects on different populations. So, personalized treatment is very important and should be considered. Thus, in future research into lysine, personalized approaches could be considered and adopted to provide better outputs for diverse populations. However, with pain being a subjective and emotional experience, variability in response is inherent in ideas related to how we view responsiveness to pain. Thus, advancing our understanding will require a more adaptable and responsive approach to the design of research and the application of knowledge.

## 8. Conclusions and Implications for Clinical Practice

In conclusion, lysine has promising potential as a novel and effective adjunct in the control of pain, This is partly attributed to its stimulatory effects on the ascending and descending pain control pathways within the central nervous system (CNS). Yet the potential utilization of lysine salts in pain control strategies still requires further investigation. This novel approach calls for a personalized treatment program that should consider individual patient conditions and characteristics. Also, a dosage and treatment schedule that ensures good clinical outcomes with minimal adverse effects should take into account drug–drug interactions, age, gender, genetics, physiological factors, and especially kidney health for cGG. Given that lysine consumption could increase the absorption of minerals and change the pharmacokinetics of several drugs, it is advised to administer cGG as a nutritional supplement with fruit/vegetable juice or water alone but never with milk. In addition, patients should be instructed to drink large amounts of water and advised of the possibility of gastrointestinal side effects, such as constipation or diarrhea, until the establishment of personalized treatment. Given the future potential of lysine salt patents, many biochemical companies are currently interested in the development and clinical evaluation of lysine salts, as well as in updating the safety profiles and identifying potential side effects of L-lysine. Unfortunately, the limited number of patents currently does not permit further comments on recent advances in lysine salt formulations. Notwithstanding, the ongoing story on the use of lysine salts in pain control strategies has stimulated reflection on the potential benefits and challenges in the development and application of lysine salts within the ever-changing landscape of clinical pain management.

## Figures and Tables

**Table 3:** Overview of lysine’s antinociceptive experiments [55,56,57,58].

Model	Animal	Lysine Dose	Effect	Notes
Formalin Test	Rats	0.5 or 2 g/kg (s.c.)	↓ second-phase paw licking	Pain is reduced without affecting movement
c-Fos Neuron Activity	Rats (spinal cord)	0.5 or 2 g/kg (s.c.)	↓ active pain neurons (c-Fos)	Indicates reduced pain signals in the spinal cord
Acetic Acid Writhing	Mice	1 g/kg (i.p.) (intraperitoneal)	Writhing is completely suppressed	Lysine blocked pain caused by acid injection
Interaction with L-Arginine	Mice	L-Arginine 3 g/kg i.p. (intraperitoneal)	Blocked lysine’s effect	Suggests nitric oxide (NO) involvement
ADMA Interaction (NO Inhibitor)	Rats	ADMA before lysine (i.v.) infusion	Reversed lysine’s effect	Further confirms that the NO pathway plays a role
Multiple-Dose Testing	Rats	0.1–5 g/kg (p.o.) (oral gavage)	5 g/kg reduced writhing significantly	Especially after 24 h of acetic acid injection
